# Six1 Promotes Proliferation of Pancreatic Cancer Cells via Upregulation of Cyclin D1 Expression

**DOI:** 10.1371/journal.pone.0059203

**Published:** 2013-03-20

**Authors:** Zhaoming Li, Tian Tian, Feng Lv, Yu Chang, Xinhua Wang, Lei Zhang, Xin Li, Ling Li, Wang Ma, Jingjing Wu, Mingzhi Zhang

**Affiliations:** 1 Department of Oncology, The First Affiliated Hospital, Zhengzhou University, Zhengzhou, People’s Republic of China; 2 Department of Neurology, The First Affiliated Hospital, Zhengzhou University, Zhengzhou, People’s Republic of China; 3 Department of Surgery, People’s Hospital of Henan Province, Zhengzhou, People’s Republic of China; Technische Universität München, Germany

## Abstract

Six1 is one of the transcription factors that act as master regulators of development and are frequently dysregulated in cancers. However, the role of Six1 in pancreatic cancer is not clear. Here we show that the relative expression of Six1 mRNA is increased in pancreatic cancer and correlated with advanced tumor stage. *In vitro* functional assays demonstrate that forced overexpression of Six1 significantly enhances the growth rate and proliferation ability of pancreatic cancer cells. Knockdown of endogenous Six1 decreases the proliferation of these cells dramatically. Furthermore, Six1 promotes the growth of pancreatic cancer cells in a xenograft assay. We also show that the gene encoding cyclin D1 is a direct transcriptional target of Six1 in pancreatic cancer cells. Overexpression of Six1 upregulates cyclin D1 mRNA and protein, and significantly enhances the activity of the cyclin D1 promoter in PANC-1 cells. We demonstrate that Six1 promotes cell cycle progression and proliferation by upregulation of cyclin D1. These data suggest that Six1 is overexpressed in pancreatic cancer and may contribute to the increased cell proliferation through upregulation of cyclin D1.

## Introduction

Pancreatic cancer is the fourth leading cause of cancer-related mortality across the globe [1]. It has often a poor prognosis due to the lack of reliable early diagnostic methods and effective treatment [2]. Therefore, there is an urgent need to improve our understanding of the molecular mechanisms of pancreatic cancer tumorigenesis and to develop effective treatment strategies.

Six1 is a mammalian homolog of the Drosophila sine oculis gene and is highly conserved from Drosophila to humans [3], [4]. It is broadly expressed in many tissues during the early development, while its expression is low or absent in most adult tissues [5]. During the early development, Six1 promotes the progenitor cell proliferation and survival through activation or repression of a diverse range of downstream target genes [3], [6]. It also plays a role in cellular migration and invasion during embryogenesis through induction of epithelial–mesenchymal transition (EMT) [7], [8]. The correct expression of this gene is critical for the development of diverse organs such as the brain, ear, eye, muscle, kidney sensory structures, and craniofacial structures [7], [9], [10]. In addition to the role of Six1 in the early development, it is often misexpressed in various tumors such as breast cancer [5], [11], Wilms’ tumors [12], rhabdomyosarcomas [13], hepatocellular carcinoma [14], ovarian cancer [15], [16], and cervical cancer [17], [18]. More importantly, the misexpression of Six1 in cancer can induce developmental programs out of context, contributing to tumor onset and progression [19], [20]. Recently, some studies have shown that overexpression of Six1 facilitates the metastasis of breast cancer through TGF-β signaling, epithelial-mesenchymal transition [21], and inducing lymphangiogenesis via upregulation of VEGF-C [22]. Inhibition of endogenous Six1 expression suppresses tumorigenesis and metastasis of hepatocellular carcinoma [23].

However, the role of Six1 in pancreatic cancer is unknown. The critical role of Six1 in the initiation and progression of numerous cancers impelled us to study the function of Six1 in pancreatic cancer. In this study, we demonstrate that Six1 is overexpressed in pancreatic cancer and correlated with advanced tumor stage. Using *in vitro* and *in vivo* functional assays, we show that forced overexpression of Six1 aberrantly promotes proliferation, contributing to pancreatic tumorigenesis. We also demonstrate that the gene encoding cyclin D1 is a direct transcriptional target of Six1 in pancreatic cancer cells. Furthermore, we show that Six1 promotes cell cycle progression and proliferation by upregulation of cyclin D1.

## Materials and Methods

### Ethics Statement

The collection of tissue samples was approved and supervised by the Research Ethics Committee of Zhengzhou University. Written informed consents were obtained from all patients who provided samples. Animal studies and xenograft tumor model were approved and supervised by Research Ethics Committee of Zhengzhou University.

### Cell Culture

Human pancreatic carcinoma cell lines PANC-1 (CRL-1469) and MIA PaCa-2 (CRL-1420) were obtained from American Type Culture Collection ATCC (Rockville, MD, USA). All cell lines were cultured in Dulbecco’s modified Eagle’s medium (Hyclone, Logan, UT, USA) supplemented with 10% fetal bovine serum (Hyclone, Logan, UT, USA), 100 units/ml penicillin, and 0.1 mg/ml streptomycin (Invitrogen, California, USA) in 5% CO_2_ atmosphere at 37°C.

### Human Tissue Samples

Primary pancreatic tumor (n = 51) and adjacent non-tumor tissues (n = 13) were collected between 2005 and 2010 from patients with resected primary pancreatic ductal adenocarcinomas at the First Affiliated Hospital of Zhengzhou University. The adjacent non-tumor tissue was obtained from a segment of the resected specimens that was the farthest from the tumor (more than 5 cm). Tissues were flash frozen immediately after surgery. No previous local or systemic treatment had been conducted on these patients before the operation. All data including age, gender, tumor size, location, stage, differentiation, perineural invasion and lymph node status were obtained from original pathology reports. Pathologic staging was updated according to current American Joint Committee on Cancer guidelines. Gene expression for Six1 was obtained for each pancreatic tumor, and all samples were mean centered. Samples were then divided into two groups for further analysis: samples in which Six1 expression was above the mean (Six1 “high”), and the remaining samples (Six1 “low”). Written informed consents were obtained from all patients and this study was approved by the Research Ethics Committee of Zhengzhou University (Zhengzhou, China).

### Plasmids Preparation and Establishing Stable Cell Lines

Human Six1 cDNA was amplified from human skeletal muscle by Reverse transcription polymerase chain reaction (RT-PCR). Human skeletal muscle tissue was obtained from the normal surrounding tissue margin of sarcoma resection specimens. The detailed reaction condition of PCR was performed according to previous report [5]. The Six1 cDNA was then subcloned into plasmid pcDNA4/TO (Invitrogen, California, USA). pCMV-cyclin D1 was obtained from Sevicebio (Wuhan, China). pcDNA4/TO-Six1 and pCMV-cyclin D1 were purified in large scale using the EndoFree Plasmid Maxi kit (Qiagen, Germany)for transfection. PANC-1 or MIA PaCa-2 cells were seeded in six-well plate and transfected with pcDNA4/TO-Six1 by using Lipofectamine 2000 reagent as recommended by the manufacturer (Invitrogen, California, USA). Twenty-four hours after transfections the cells were passaged and selected using 100 µg/ml Zeocin for 2 weeks, and then got the pcDNA4/TO-Six1 stable cell lines.

### RNA Interference

For small interfering RNA (siRNA)-mediated down regulation of Six1 or cyclin D1, the following siRNA sequences were purchased from Ribobio Company, Guangzhou, P.R.China). The sequences of the Six1 targeted siRNA are 5′-AGAACGAGAGCGUACUCAA-3′ or 5′-GGGAGAACACCGAAAACAA-3′; the sequence of the cyclin D1 targeted siRNA is 5′-GTTCATTTCCAATCCGCCC-3′ or 5′-GCCGAGAAGUUGUGCAUCUUU-3′. PANC-1 and MIA PaCa-2 cells were seeded in 6-well plates, grown to 50% confluency and transfected with siRNA or scramble control siRNA (provided by Ribobio Company, Guangzhou, China) duplexes using lipofectamine 2000 (Invitrogen, California, USA) according to the manufacturer’s recommendations. 20 nM siRNAs was used per well and cells were incubated for another 48 to 72 hours before being harvested for protein extraction and immunoblot analysis.

### Quantitative Real-time RT-PCR

Total RNA was isolated using the RNeasy mini kit according to the manufacturer’s instructions (Qiagen, Germany). The quantity and purity of the isolated RNA was quantified with a NanoDrop 1000 spectrophotometer (Thermo Scientific, Wilmington, DE, USA) at wavelengths of 230, 260, and 280 nm. Only RNA samples with a 260/280 ratio from 1.9 to 2.1 and a 260/230 ratio from 2.0 to 2.5 were acceptable for further analysis. The integrity of total RNA was assessed by visualization of the 18S and 28S ribosomal RNA patterns in a denaturing agarose gel (Qiagen, Germany). And the sharp, clear 28S and 18S rRNA bands and a 28S/18S ratio of two are considered to be good quality RNA. The first-strand cDNA was synthesized using the SuperScript® III First-Strand Synthesis System according to the manufacturer’s instructions (Invitrogen, California, USA). Quantitative PCR was done using SYBR Green dye on an Applied Biosystems 7300 Real-time PCR system (Applied Biosystems, Foster City, CA). Briefly, 10 µL of RNA/primer mixture was prepared in a sterile 0.2 mL tube as follows: 0.5 µg of total RNA, 1 µL of Oligo(dT)_20_ (50 µM), 1 µL of 10 mM dNTP mix and DEPC-treated water to a final volume of 10 µL. The mixture was then incubated at 85°C for 5 min and chilled on ice for at least 1 minute before adding 10 µl of cDNA Synthesis Mix (2 µL of 10×RT Buffer, 4 µL 25 mM MgCl_2_, 2 µL 0.1 M DTT, 1 µL RNaseOUT™ and 1 µL SuperScript® III Reverse Transcriptase). The 20 µl reverse transcription reaction was incubated at 50°C for 30 min, followed by 85°C for 5 min. RNA template was removed by adding 1 µl of RNase H and incubating at 37°C for 20 min. Quantitative PCR was done using SYBR Green dye on an Applied Biosystems 7300 Real-time PCR system (Applied Biosystems, Foster City, CA). The quantitative PCR was performed with 12.5 µL of 2×SYBR Green PCR mastermix (Tiangen Biotech, Beijing, China), 1 µL of template cDNA, 200 nM each amplification primer and DEPC-treated water to a final volume of 25 µL. The reactions were incubated in a 96-well optical plate at 95°C for 5 min, followed by 40 cycles of 94°C for 15 s and 60°C for 30 s. The threshold cycle (C_T_) data was determinate using default threshold settings. The C_T_ is defined as the fractional cycle number at which the fluorescence passes the fixed threshold. The real-time PCR data was analysed by comparative C_T_ method [24]. All reactions were run in duplicate and sterile water was tested along with the sample as negative control. Gel electrophoresis was performed to confirm the exclusive amplification of the expected PCR product. Primer sets used were as follows: for gene Glyceraldehyde 3-phosphate dehydrogenase (GAPDH), 5′-GGAGCGAGATCCCTCCAAAAT-3′ and 5′-GGCTG TTGTCATACTTCTCATGG-3′; for Six1, 5′-AAGGAGAAGTCGAGGGG TGT-3′ and 5′-TGCTTGTTGGAGGAGGAGTT-3′; for cyclin D1, 5′-GCTGCGAAGT GGAAACCATC-3′ and 5′-CCTCCTTCTGCACACATTTGAA-3′.

### Analysis of Microarray Data

Oncomine Cancer Microarray database [25] (http://www.oncomine.org) were used to study gene expression of Six1 in human pancreatic cancer samples as we previously described [26]. Gene expression data were also obtained from NCBI Gene Expression Omnibus (GEO) database (accession numbers GSE15471, GSE32676 and GSE28735; http://www.ncbi.nlm.nih.gov/geo) [27]–[29]. Expression data for Six1 and cyclin D1 were log-transformed, median centered per array, and the standard deviation was normalized to one per array.

### Western Blot Analysis

Western blot analysis was performed as we previously described [30]. Briefly, cells were lysed in cold lysis buffer [Tris-HCl pH 7.5, 150 mM NaCl, 1 mM EDTA, 1 mM MgCl_2_, 0.5% Triton X-100, phosphatase inhibitor mixture (1 mM NaF, 1 mM Na_3_VO_4_ and 1 mM β-glycerophosphate), and a protease inhibitor mixture (1 mM PMSF, 2 µg/ml aprotinin, 1 µg/ml leupeptin and 1 µg/ml pepstatin A)]. Lysates were clarifed by centrifugation at 10,000×g for 20 min. Proteins (10–25 µg) were resolved on SDS-PAGE, transferred onto nitrocellulose membranes (Amersham Biosciences, Piscataway, NJ, USA). The membrane was blocked in TBS-T buffer (20 mM Tris-Hcl, pH 7.5, 150 mM Nacl and 0.05% Tween-20) containing 5% (w/v) non-fat milk at room temperature for 1 hour and then probed with antibodies for Six1, cyclin A, cyclin B1, cyclin E, GAPDH (all from Santa Cruz Biotech, Santa Cruz, CA, USA), Phospho-Rb (#8516, Cell Signaling Technology), Rb (#9313, Cell Signaling Technology) and cyclin D1 (#2926, Cell Signaling Technology) at 4°C overnight. Detection was performed with the SuperSignal West Femto Maximum Sensitivity Substrate Trial Kit (Pierce, Rockford, IL, USA). The band images were digitally captured and quantified with a FluorChem FC2 imaging system (Alpha Innotech, San Leandro, CA, USA).

### MTT and Cell Count Assay

One thousand cells were seeded into 96-well culture plates and incubated at 37°C for different periods of time. Then the 20 µL of MTT (tetrazolium bromide, 5 mg/mL, GE Healthcare) was added to each well and incubated for 4 h at 37°C. The culture medium was removed and 150 µL of DMSO was added to solubilize the crystals for 20 min at room temperature and the absorbance at 570 nm was read by an ELISA plate reader (Model 680, Bio-Rad, CA). For cell counting, cells plated in 6-well plates were incubated at 37°C for different periods of time and then removed by trypsinization, and the number of viable cells was counted in a hemocytometer with the use of trypan blue staining. Every sample was measured in triplicate and repeated three times.

### BrdU Incorporation

Cells were exposed to 10 µM BrdU (BD Biosciences, Mountain View, CA) for 30 min and fixed in 70% ethanol, and then washed with PBS, resuspended, and incubated with 4 N HCL and 0.5% Triton X-100 for 30 min at room temperature. After washing with PBS, cells were neutralized with 0.1 M sodium borate before being labeled with FITC-conjugated BrdU antibody (BD Biosciences, Mountain View, CA) and incubated with 50 µg/ml propidium iodide (Sigma Chemical Company, St. Louis, MO) according to the manufacturer’s protocol before being analyzed by a Becton Dickinson FACStar Plus flow cytometer.

### Colony Formation Assay

Six hundred cells were seeded into a 6-well plate. After 14 days, cells were stained by 0.5% crystal violet (Sigma-Aldrich, St. Louis, MO, USA) in methanol for 10 min. Colonies (more than 50 µm diameter) were counted directly on the plate. Statistical significance was calculated from at least three independent experiments.

### Reporter Assays

Panc-1 cells were plated in 24-well plates at 100,000 cells/well and allowed to grow under normal culture conditions. At 80% conﬂuency, cells were transfected with expression vectors (Six1 or empty vector), the full-length human cyclin D1 promoter (–1745D1/LUC, –1745 to +155) [31] and renilla reniformis luciferase expression vector pRL-TK (Promega, Madison, WI, USA). The molar ratio of pRL-TK to pGL3-reporter vector was 1: 10. Then the luciferase activities were measured by using the Dual Luciferase Reporter Assay System (Promega, Madison, WI, USA) according to the manufacturer’s instructions. Cells were lysed after 48 h with 500 µl 1×Passive Lysis Buffer (Promega) per well, incubated for 20 min at room temperature and cell debris removed by centrifugation. 50 µl supernatant was mixed with 50 µl of Dual-Glo reagent I (Promega) and fireﬂy luciferase luminescence measured in a GloMax® 20/20 Luminometer (Promega, Madison, WI, USA). 50 µl Dual-Glo reagent II (Promega) was added and Renilla luciferase luminescence determined. Relative light units were calculated as Renilla/fireﬂy luminescence from triplicate wells and normalized to the negative control.

### Chromatin Immunoprecipitation (ChIP) Assay

The ChIP assays were performed by using a ChIP assay kit (Upstate Biotechnology, Inc., Lake Placid, NY) as suggested by the manufacturer. Brifely, cells (15 cm culture dish, approximately 1.0×10^7^ cells) were fixed with 1% formaldehyde for 10 min at room temperature, followed by washing twice with 20 ml of cold 1×PBS and harvested by scraping. After centrifugation, cell pellets were lysed in 1 mL of SDS Lysis Buffer containing Protease Inhibitor Cocktail II. Cell lysate was sonicated on wet ice four times for 15 seconds each time at intervals of 15 seconds to obtain chromatin fragments of about 200–1000 bp nucleotides. Centrifuged at 12,000×g at 4°C for 10 min to remove insoluble material, and then removed supernatant to fresh microfuge tubes in 100 µL aliquots. Each 100 µL supernatants were diluted with 900 µL of ChIP dilution buffer and preincubated with Protein G agarose at 4°C for 1 hour, and then pelleted agarose by brief centrifugation and removed 10 µL (1%) of the supernatant as Input. Supernatants were incubated at 4°C overnight with 5 µg of Six1 (sc-9709, Santa cruz), RNA Polymerase II antibody (Pol II, sc-56767, Santa cruz) and normal IgG, (sc-2028, Santa cruz) incubated overnight at 4°C with rotation, and then added 60 µL of Protein G Agarose to each tube and incubated for 1 hour at 4°C with rotation. After centrifugation, removed supernatant and washed the Protein G Agarose-antibody/chromatin complex by resuspending the beads in 1 mL each of Low Salt/High Salt/LiCl Immune Complex Wash Bufferwash buffer for one time, followed by washing twice with TE buffer. After the final washing, the immunoprecipitates were eluted and reverse cross-linked by incubation overnight at 65°C in elution buffer. DNA was then purified with a PCR purification kit (Qiagen, Hilden, Germany). Immunoprecipitated DNAs were analyzed by quantitative real-time PCR using the following primers: cyclin D1 promoter (−1189 to −985), 5′-AGGAACCTT CGGTGGTCTTG-3′ and 5′-CCTTGACCAGTCGGTCCTTG-3′; cyclin D1 promoter (−348 to −190), 5′-CTCAGGGATGGCTTTTGGGC-3′ and 5′-ACTCCCCTGTAGT CCGTGTG-3′; the positive control GAPDH gene proximal promoter, 5′-TACTAGC GGTTTTACGGGCG-3′ and 5′-TCGAACAGGAGGAGCAG AGAGCGA-3′.

### Mouse xenograft Model

Female BALB/c (6–8 weeks old) athymic nude mice were purchased from Experimental Animal Center of Henan province (Zhengzhou, China). The mice were housed at five/cage in microisolator units under humidity and temperature controlled conditions with 12-hour light-dark cycles. There were fed with a standard sterile laboratory diet (Zhengzhou University, Zhengzhou, China) at least three days before use. Animal health was examined prior to tumor implantation and randomization to ensure that only animals without any symptoms of disease were selected to enter testing procedures. During the experiments, animals were monitored twice daily regarding tumor burden, general condition, food and water supply. The mice were randomly distributed into two groups and subcutaneously injected in the flank regions with 5.0×10^6^ cells in 0.1 mL of PBS. Group 1 (vector control) was injected with PANC-1 cells which was stablely transfected with pcDNA4/TO; group 2 (Six1) was injected with PANC-1 cells which was stablely transfected with pcDNA4/TO-Six1. When the needle was injected below the skin, held the needle parallel to the animal’s body to avoid puncturing underlying structures and aspirated the syringe to ensure that the needle has not entered a blood vessel. The tumor size was measured every 5 days with calipers. The tumor volume was calculated with the formula: (Length × Width^2^)/2. Five weeks following implantation, a tumor began to appear at the site of implantation with 400 to 800 mm^3^ in volume. Mice were euthanized by asphyxiation in a CO_2_ chamber and tumors excised using standard forceps, scissors, and surgical blades. All procedures were conducted in accordance to Animal Care and Use Committee guidelines of Zhengzhou University.

### Statistical Analysis

All data were expressed as mean ± s.e.m. Between groups and among groups comparisons were conducted with Student t test and ANOVA, respectively. Mann-Whitney U test is used for nonparametric variables. The association of Six1 mRNA expression and clinicopathological characteristics including age, gender, tumor size, location, stage, differentiation, perineural invasion and lymph node status was analysed by either the Chi-square or Fisher’s two-tailed exact test. The Spearman rank correlation test was assessed to verify the association between expression levels of Six1 and cyclin D1 on log-transformed values. Statistical analysis was performed using GraphPad Prism software version 4.0 (PRISM4) (GraphPad Software Inc, LaJolla, CA), and *P*<0.05 was considered significant.

## Results

### Six1 is Overexpressed in Pancreatic Cancer and Correlated with Advanced Tumor Stage

**Figure 1:**
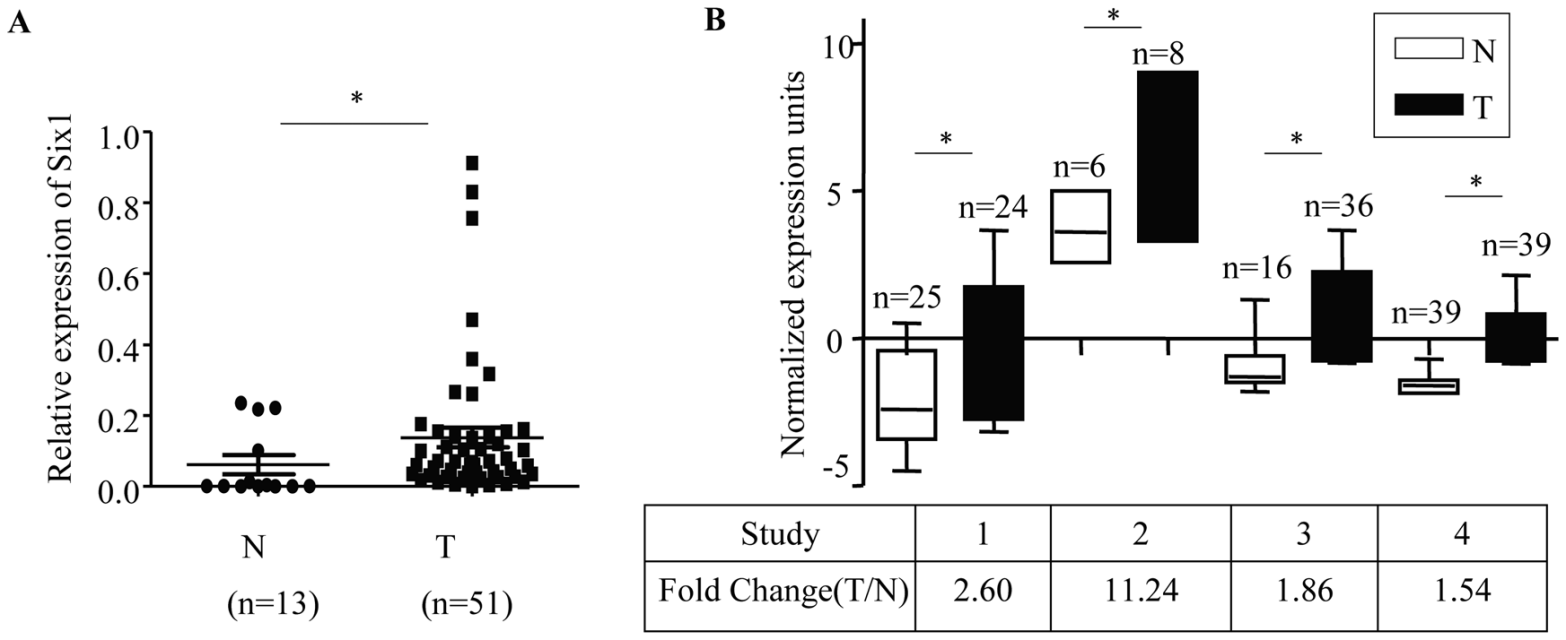
Expression of Six1 in pancreatic cancer. (A) The relative mRNA expression level of Six1 was determined by quantitative Real-time RT-PCR in 51 pancreatic ductal adenocarcinomas and 13 adjacent non-tumor pancreatic tissue samples. Results were normalized to the expression level of GAPDH mRNA in each sample. *P<0.05. (B) ONCOMINE database was used to analyze previously published microarray data. Levels of Six1 mRNA are increased in pancreatic cancer when compared with adjacent non-tumor pancreatic tissue. All results, including *p*-values, were calculated using ONCOMINE data. N, adjacent non-tumor pancreatic tissue; T, pancreatic ductal adenocarcinomas; *P<0.05.

To investigate the role of Six1 in pancreatic cancer development, we tested the expression of Six1 in 51 pancreatic ductal adenocarcinomas and 13 adjacent non-tumor pancreatic tissue samples using quantitative Real-time RT-PCR. The expression of Six1 mRNA in pancreatic ductal adenocarcinomas samples was significantly higher than in the adjacent non-tumor pancreatic tissues after normalization using GAPDH (*P*<0.01) (Figure 1A). We further investigated the expression of Six1 by querying the ONCOMINE database. In four microarray expression studies [28], [32]–[34], the expression of Six1 mRNA is significantly higher in pancreatic cancer tissues than in the adjacent non-tumor pancreatic tissues; the mRNA levels increase between 1.5- and 11.2-fold (Figure 1B). It may be noted that non-tumorous tissue adjacent to malignant pancreatic cancer is not the normal pancreatic tissue and therefore those data may not reflect truly the expression of normal pancreatic tissue.

The gene expression pattern of Six1 in pancreatic ductal adenocarcinomas was compared with their clinicopathological characteristics (Table 1). Overexpression of Six1 mRNA was significantly correlated with tumor stage (*P* = 0.018). About 67% (18 of 27) of advanced stage (III & IV) of pancreatic ductal adenocarcinomas were found to overexpress Six1. There is no significant association between Six1 and age, gender, tumor size, location, differentiation, perineural invasion and lymph node status (all *P*>0.05; Table 1).

**Table 1:** Clinicopathological characteristics of patients according to the expression of Six1.

Characteristics	Six1 Low(n = 25)	Six1 High(n = 26)	*P* value
**Age (years)**			0.210
<60	12	17	
≥60	13	9	
**Gender**			0.657
Female	10	12	
Male	15	14	
**Tumor size**			0.683
<4 cm	11	11	
≥4 cm	14	15	
**Location**			0.688
Head	14	16	
Body/rear	11	10	
**Stage**			0.018
I & II	16	8	
III & IV	9	18	
**Lymph node status**			0.091
Negative	9	4	
Positive	16	22	
**Perineural invasion**			0.349
Negative	8	5	
Positive	17	21	
**Differentiation**			0.558
Well	7	5	
Moderate	8	12	
Poor	10	9	

### Six1 Promotes Growth of Pancreatic Cancer Cells *in vitro* and *in vivo*

**Figure 2:**
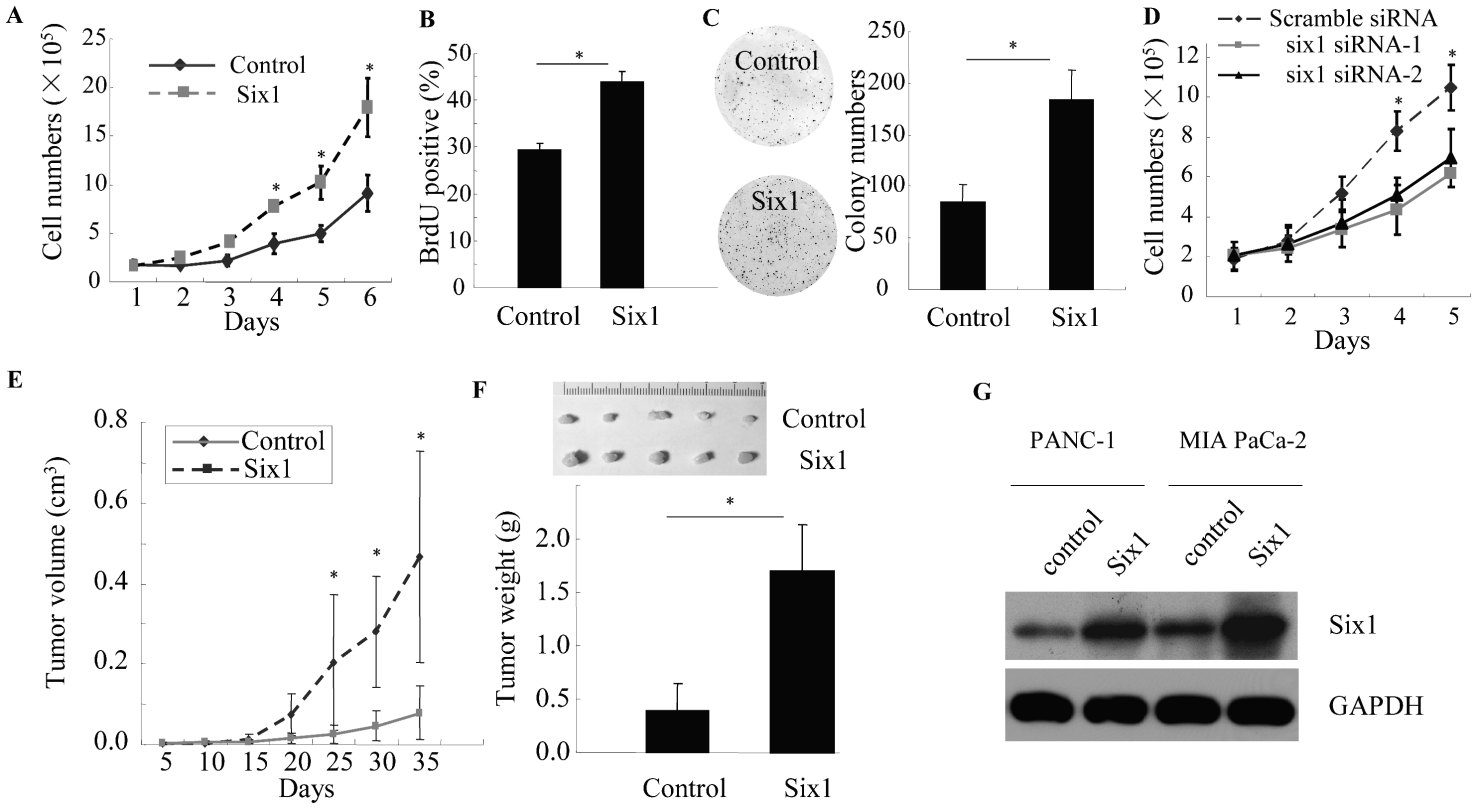
Six1 increases pancreatic cancer cell proliferation *in vitro* and *in vivo*. Cell numbers (A), percentage of BrdU positive cells (B) and number of colonies (C) of PANC-1 cells stably transfected with either Six1 or control plasmids. Solid line, control; dashed line, Six1. D, Number of PANC-1 cells transfected with Six1 siRNA and scramble control. Solid line, scramble control siRNA; dashed line, Six1 siRNA. *E, Tumor size of subcutaneous xenografts measured within 5-day interval. The tumor volume was calculated using the formula: (Length × Width^2^)/2. F, Representative subcutaneous tumor xenografts generated in mice* 5 weeks *after inoculation (upper panel), and* the weight of the tumors (*lower panel*). G, The expression of Six1 in both PANC-1 and MIA PaCa-2 cells was determined by western blot analyses. GAPDH was used as an internal control. All experiments were performed in triplicate; bars, s.e.m.; *P<0.05.

In order to explore the functional role of Six1 in pancreatic cancer, we examined the effect of Six1 overexpression on the proliferation of both PANC-1 and MIA PaCa-2 cells. The cells were stably transfected with either pcDNA4/TO-Six1 or control vector plasmids. The western blot analysis confirmed that the expression of Six1 increased in both PANC-1 and MIA PaCa-2 cells transfected with pcDNA4/TO-Six1, in comparison with those transfected with control vector (Figure 2G and Figure S1A).

Cell proliferation was assessed by direct cell count, MTT *growth assay*, BrdU incorporation, and colony formation assay. Both MIA PaCa-2 and Panc-1 cells were used. According to the cell count results, proliferation rate of the cells with overexpressed Six1 was significantly higher than that of the control cells; more Six1-transfected cells were observed at 4, 5, and 6 days after plating (*P<0.05, Figure 2A). *MTT growth assays* showed that the cell growth rate significantly increased after overexpression of Six1 (*P<0.05, Figure S1B). In the BrdU incorporation assay, the numbers of BrdU positive cells in control and pcDNA4/TO-Six1 group were 29.3% ±1.7% and 44.0% ±1.1%, respectively (*P<0.05, Figure 2B). In colony formation assay for PANC-1 the numbers of colonies formed for vector control and pcDNA4/TO-Six1 group were 84±16 and 184±28, respectively (*P<0.05, Figure 2C). We obtained similar results *for* MIA PaCa-2 cells in colony formation assay (*P<0.05, Figure S1C).

To test whether Six1 is required for proliferation of pancreatic cancer cells, we silenced Six1 in both PANC-1 and MIA PaCa-2 cells using siRNA method. The cell count assay demonstrated that the number of Six1-knockdown cells was significantly lower than the number of both PANC-1 and MIA PaCa-2 cells transfected with scrambled control (Figure 2D and Figure S1D). Inhibition of endogenous Six1 in MIA PaCa-2 cells resulted in approximately 1.8-fold decrease of BrdU positive cell rates, compared to the scrambled control group. (Scrambled siRNA vs. Six1 siRNA-1, 43% vs. 26%; Scrambled siRNA vs. Six1 siRNA-2, 43% vs. 23%; *P<0.05, Figure S1E). Similar results were obtained in the PANC-1 cells (Figure S1F). The silencing of Six1 expression was confirmed after Six1 siRNA treatments (20 nM for 72 hours) by both quantitative PCR and Western blot analysis (Figure S2). Taken together, these results indicate that Six1 promotes cell proliferation of pancreatic cancer cells.

To confirm the above findings, an *in vivo* tumor xenograft model was used. PANC-1 cells stably overexpressing Six1 or control cells were injected subcutaneously into two groups of nude mice (n = 5). As expected, the tumor growth curve showed that tumors derived from Six1 group grew more rapidly than those from the control group. Five weeks after injection, the tumor volume of Six1 group was 0.47±0.26 cm^3^, whereas the tumor volume of control group was 0.08±0.06 cm^3^ (*P<0.05, Figure 2E). Moreover, the mean tumor weight at the end of the experiment was significantly higher in the group overexpressing Six1 (1.69±0.44 g) compared to the control group (0.39±0.25 g; *P<0.05, Figure 2F). These results demonstrate that Six1 increases the growth of pancreatic cancer *in vivo*.

### Six1 Transcriptionally Activates the Gene Encoding Cyclin D1 in Pancreatic Cancer Cells

**Figure 3:**
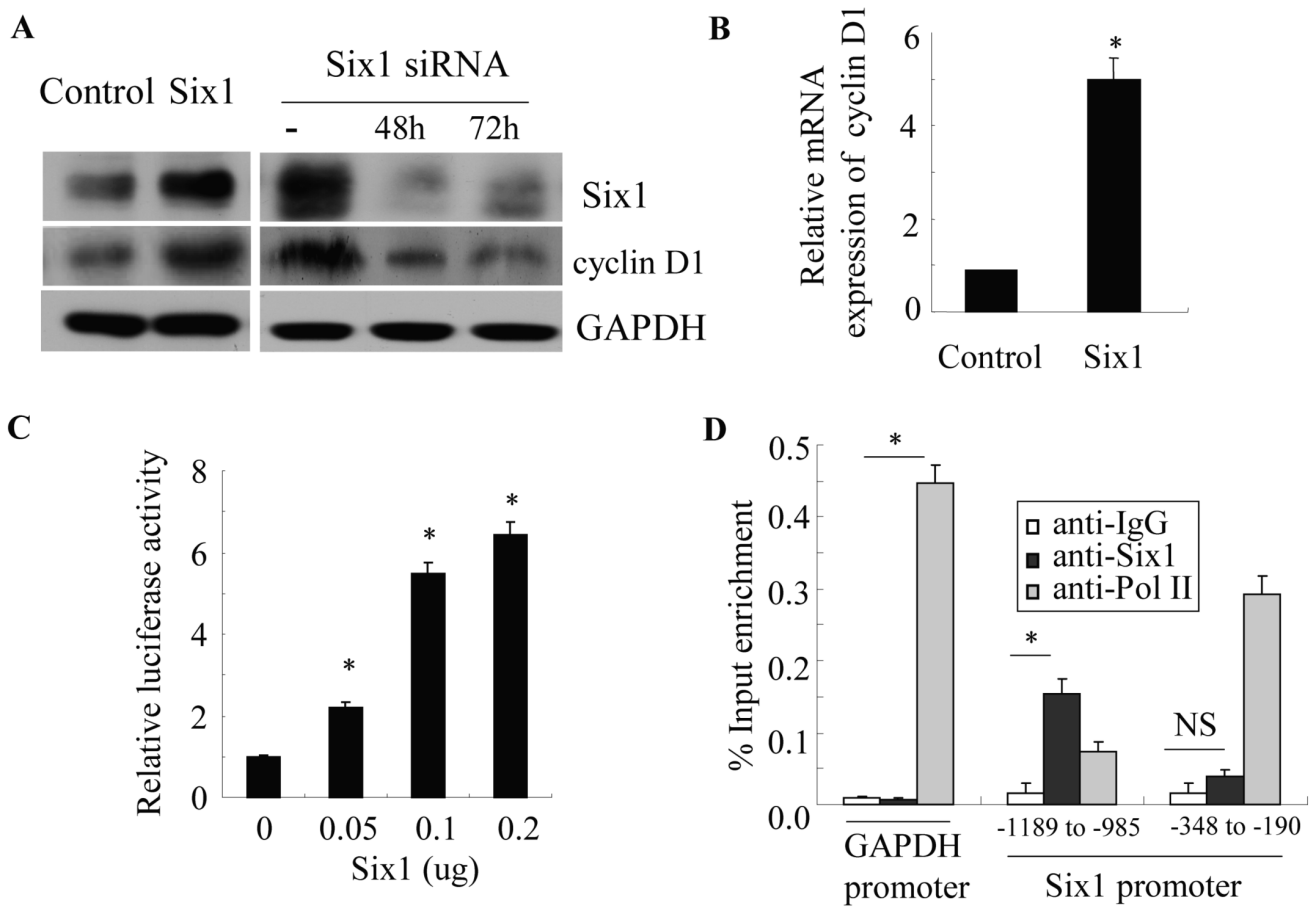
Six1 transcriptionally activates the gene encoding cyclin D1 in pancreatic cancer cells. A and B, The effect of Six1 on the expression of cyclin D1 protein (A) and mRNA (B) was examined in PANC-1 cells by western blot analyses. GAPDH was used as an internal control. C, cyclin D1 promoter activity was shown to be responsive to increased amounts of a Six1 expression vector, using a luciferase reporter. D, the physical interaction of Six1 to the cyclin D1 promoter in chromatin of PANC-1 cells was shown using a ChIP-qPCR assay. The recruitment of Pol II to the GAPDH proximal promoter was used as a positive control, and the negative control is normal IgG, which controls for the non-specific immunoselection of chromatin by immunoglobulins. Soluble chromatin was prepared and immunoprecipitated with the indicated antibodies. The final DNA extractions were amplified using three pairs of primers as indicated. It showed that Six1 could bind to the −1189 to −985 region of the cyclin D1 promoter but not to the irrelevant −348 to −190 region. All experiments were performed in triplicate; NS, not significant; *P<0.05.

It has been reported that Six1 is a cell cycle regulator and affects the cell cycle progression both in normal development and in cancer [3], [5], [20], [35]. To study underlying molecular mechanisms by which Six1 promotes cell growth of pancreatic cancer cells, we analyzed several cell cycle regulators and found that cyclin D1 protein levels were significantly higher in PANC-1 and MIA PaCa-2 cells transduced with Six1, whereas the cells with Six1 knockdown showed a reduced cyclin D1 expression (Figure 3A and Figure S1A). Six1 is a transcription factor that acts as one of the master regulators of development. To determine whether Six1 induced cyclin D1 expression at the transcription level, we analyzed cyclin D1 expression by quantitative PCR analysis. We found that cyclin D1 mRNA level increased at least 4-fold in Six1 transduced PANC-1 cells (*P<0.05, Figure 3B). We next asked whether Six1 could directly regulate the activity of cyclin D1 promoter. PANC-1 cells were transfected with the human cyclin D1 promoter reporter in the presence of increasing concentrations of human Six1-expression plasmid. The data showed that Six1 significantly enhanced the luciferase activity of cyclin D1 promoter reporter in a dose-dependent manner (*P<0.05, Figure 3C). Next, we performed ChIP analysis to determine whether Six1 binds to the cyclin D1 promoter in chromatin. Normal IgG bound to cyclin D1 promoter was used as negative control. RNA polymerase II antibody-immunoprecipitated DNA was amplified with primers of GAPDH promoter to serve as positive control. Figure 3D shows that Six1 can bind to the cyclin D1 promoter between -1189 and -985, a region containing the two MEF3-like motifs (TCAGCTTTC and TAATATTTC) [36]. In contrast, an irrelevant sequence near the transcriptional start site (−348 to −190) did not bind Six1. To summarize, Six1 positively regulated cyclin D1 expression at both mRNA and protein level, suggesting that cyclin D1 is an *in vivo* target of Six1 in pancreatic cells.

### Six1 Expression Significantly Correlates with Cyclin D1 in Human Pancreatic Cancer

**Figure 4:**
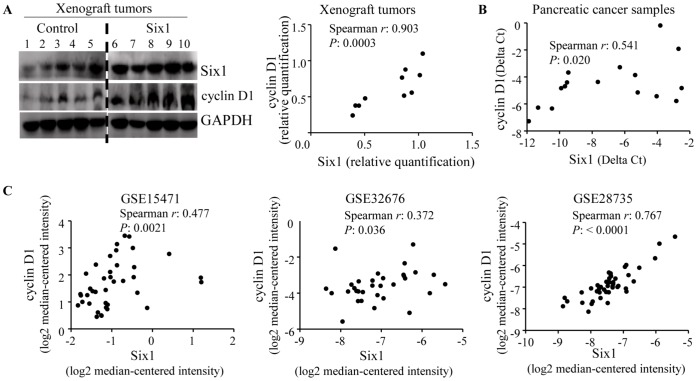
Six1 correlates with cyclin D1 in human pancreatic cancer. A, Six1 levels correlate with cyclin D1 in the xenograft tumors from PANC-1 cells stably transfected with either Six1 or control plasmids as determined by western blot and then quantified by Fluorchem computer analysis (r = 0.903, *P* = 0.0003). B, the correlation between the two in 27 advanced stage (III & IV) of human pancreatic cancer samples as determined by qRT-PCR (r = 0.541, *P* = 0.020). C, the correlation between the two in three other independent pancreatic cancer microarray datasets (GSE15471: r: 0.477, *P*: 0.0021; GSE32676: r: 0.372, *P*: 0.036; and GSE28735: r: 0.767, *P*<0.0001). Correlation between the two was quantified by Spearman’s rank correlation. *P<0.05.

To determine whether both Six1 and cyclin D1 are coordinately expressed in pancreatic cancer, we examined their protein expression in the xenograft tumors from PANC-1 cells stably transfected with either Six1 or control plasmids. The result showed that they were coordinately expressed in the xenograft tumors (r = 0.903, *P* = 0.0003; Figure 4A and r = 0.721, *P* = 0.023; Figure S3). Next, analysis of 27 advanced stage (III & IV) of human pancreatic cancer samples for Six1 and cyclin D1 expression demonstrated a statistically significant correlation between the two (r = 0.541, *P* = 0.020; Figure 4B). Furthermore, we examined the expression of Six1 and cyclin D1 in three other independent pancreatic cancer microarray datasets and all of them showed a significant correlation between the two (GSE15471: r: 0.477, *P* = 0.0021; GSE32676: r: 0.372, *P* = 0.036; and GSE28735: r: 0.767, *P*<0.0001; Figure 4C). We also examined the correlation of Six1 with various other cyclins in PANC-1 and MIA PaCa-2 cells by Western blot. It showed that ectopic Six1 expression in these cells up-regulated expression of two cyclins, cyclin D1 and cyclin A, but not cyclin B1 or cyclin E (Figure S4). Taken together, these results suggest that Six1 may also promote cyclin D1 expression in human pancreatic cancer.

### Six1 Regulates Pancreatic Cancer Cell Growth and Cell Cycle Progression through Cyclin D1

**Figure 5:**
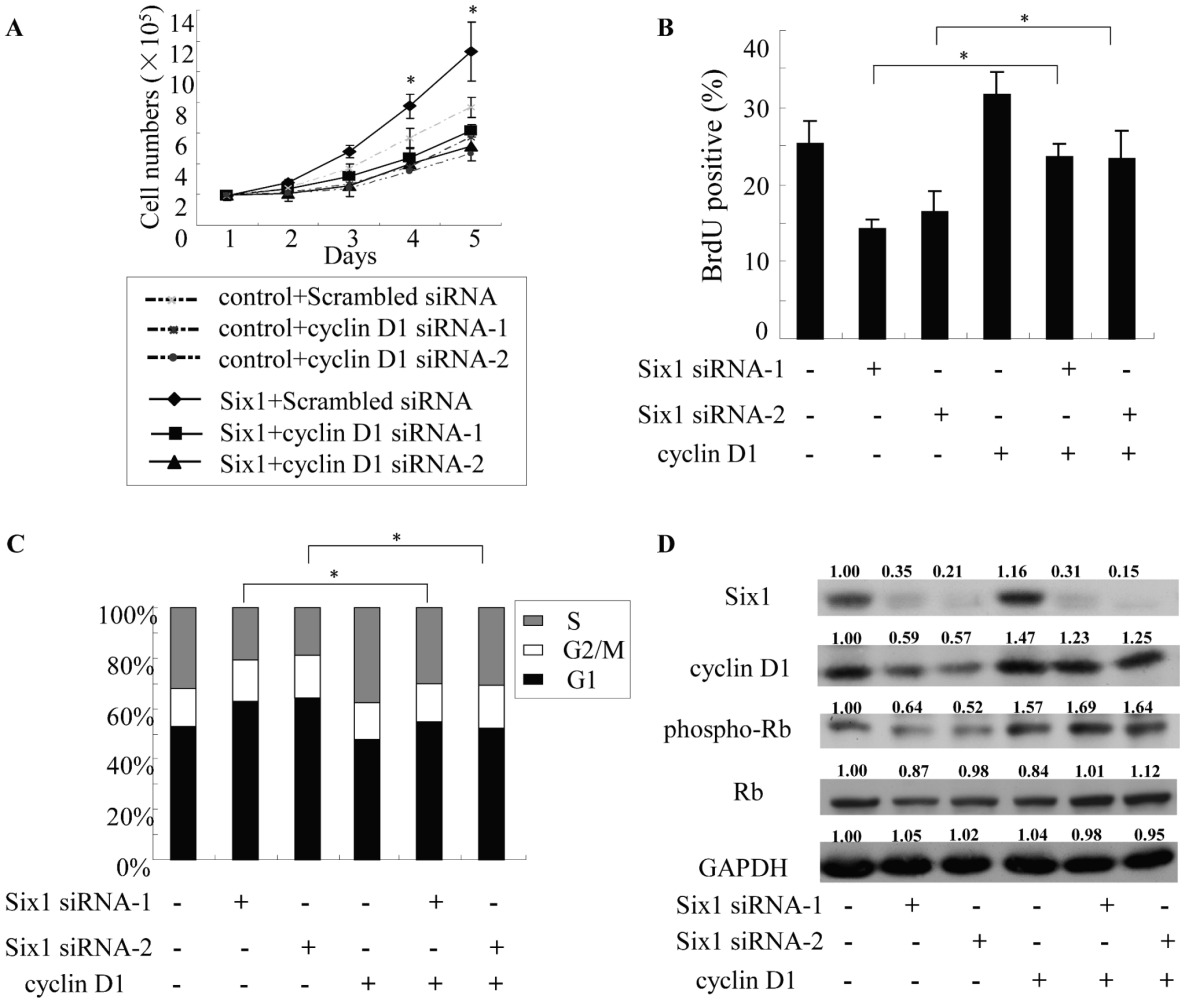
Six1 regulates pancreatic cancer cell growth and cell cycle progression through cyclin D1. A, Knockdown of cyclin D1 decreases proliferation of PANC-1 cells transduced with Six1. B, C, and D, overexpression of cyclin D1 rescued the inhibitory effect of Six1 silencing on the growth of PANC-1 cells, which was examined by BrdU incorporation assay (B) and flow cytometry analyses (C). The phosphorylation levels Rb (phospho-Rb) was also examined by western blot analyses. The expression of proteins was quantified using the expression of GAPDH as calibrator and normalized against the control group value (D). All the experiments were performed in triplicate; bars, s.e.m.; *P<0.05.

To determine whether Six1 promotes growth of pancreatic cancer through upregulation of cyclin D1, cyclin D1 siRNA was used to transfect PANC-1 cells stably transduced with Six1. According to the cell count results, knockdown of cyclin D1 significantly attenuated the growth advantage conferred by Six1 in PANC-1 cells (Figure 5A). We next tested whether overexpression of cyclin D1 could rescue the inhibitory effect of Six1 silencing on the growth of PANC-1 cells. The BrdU incorporation assay showed that knockdown of endogenous Six1 dramatically inhibited the proliferation of PANC-1 cells; and the overexpression of cyclin D1 rescued the inhibitory effect of Six1 silencing (Figure 5B). We next assessed the effect of Six1 on the cell cycle progression. Cell cycle analyses showed that knockdown of Six1 reduced the cell proliferation index (PI) from 47% to 33% in PANC-1 cells (*P<0.05, Figure 5C). However, the introduction of cyclin D1 increased cell PI from 33% to 51% (*P<0.05, Figure 5C) and decreased the G1 phase cells from 67% to 49% after co-transfection with Six1 siRNA and cyclin D1 plasmids (*P<0.05, Figure 5C). We also showed that silencing of Six1 reduced the expression of cyclin D1 and decreased phosphorylation levels of Rb (phospho-Rb). However, forced overexpression of cyclin D1 was alleviated the decrease in phospho-Rb levels in PANC-1 cells (Figure 5D). These data suggest that Six1 promotes cell growth and cell cycle progression in pancreatic cancer cells, at least in part through upregulation of cyclin D1.

## Discussion

Six1 is aberrantly expressed in numerous human tumors such as *breast cancer, Wilms’ cancer, and* rhabdomyosarcomas [5], [12], [13]. However, the expression of Six1 in pancreatic cancer has not been examined in detail. To investigate whether Six1 is deregulated in pancreatic cancer, the mRNA levels of Six1 were examined in this study. Our results showed that Six1 was overexpressed in pancreatic cancer in comparison to the adjacent non-tumor pancreas tissues. Furthermore, we queried the ONCOMINE database to confirm gene expression levels of Six1 in pancreatic cancer. In agreement with our study, four independent datasets in ONCOMINE database showed that the expression of Six1 is upregulated in pancreatic cancer. Our results complement the previous studies and further suggest that the upregulation of Six1 might contribute to pancreatic cancer tumorigenesis. However, the clinical significance of Six1 overexpression in pancreatic cancer tissues and the prognostic value of the data need to be confirmed by future studies.

The development of tumors may involve a dysregulation of oncogenes or tumor suppressor genes [37]. Some studies have reported that Six1 is oncogenic in mammary epithelial cells, promoting proliferation and genetic instability [8], [19]. Moreover, Six1 overexpression in ovarian cancer promotes the proliferative phenotype of the tumor cells [15]. In this study, we demonstrated that forced overexpression of Six1 enhanced the proliferation and colony formation of pancreatic cancer cells, while knockdown of endogenous Six1 significantly reduced the growth rate of these cells. Our results suggested that Six1 might play an essential role in tumor proliferation. Moreover, our *in vivo* xenograft tumorigenesis model also confirms that upregulation of Six1 increases the growth rate of pancreatic cancer in nude mice. These results suggested that Six1 might function as an oncogene contributing to tumorigenesis of pancreatic cancer. This is in line with the previous reports that upregulation of Six1 promotes tumorigenesis in breast cancer, ovarian cancer and hepatocellular carcinoma [15], [19], [23]. All these data suggest that Six1 may participate in oncogenic regulation in many cancers.

Cyclin D1, the regulator of the G1/S phase transition of the cell cycle, plays a key role in promoting cell proliferation [38]. It is upregulated in a variety of cancers, including pancreatic cancer, and plays a critical role in tumor initiation and progression [39]. In this study, we found that cyclin D1 was required for Six1-induced cell proliferation and tumor growth in pancreatic cancer. Firstly, we showed that Six1 induced cyclin D1 expression and directly regulated cyclin D1 promoter activity. These results are consistent with the previous report that Six1 transcriptionally activates Ezrin and cyclin D1 in rhabdomyosarcoma tumor cells [35]. Secondly, our data demonstrated that Six1 expression significantly correlates with cyclin D1 in human pancreatic cancer. Thirdly, the growth advantage conferred by Six1 in pancreatic cancer cells is cyclin D1-dependent. Six1 is known to control transit through the cell cycle. It actively promotes tumorigenesis of breast cancer by reactivation of Cyclin A1, and c-myc is transcriptionally regulated by Six1 in rhabdomyosarcoma tumor cells [35]. Taken together, these results suggest that Six1 might have a critical role in tumorigenesis through its function in cell cycle regulation.

To summarize, our study demonstrates that Six1 is overexpressed in pancreatic cancer, leading to an increase in cancer cell growth and cell cycle progression through upregulation of cyclin D1. It not only yields a better understanding of the role of Six1 in pancreatic cancer, but also paves the way for novel and powerful anticancer therapeutics. In addition, the alterations in Six1 expression become progressively worse with increasing stage of pancreatic cancer, suggesting its potential use as a new prognostic marker. Further studies are needed to define the clinical significance and prognostic value of Six1 expression in pancreatic cancer.

## Supporting Information

Figure S1 **Six1 promotes the growth of PANC-1 and MIA PaCa-2 cells.** A, Western blot analysis of the expression of Six1 and cyclin D1 in MIA PaCa-2 cells. B and C, *MTT growth assays and* colony formation assay of the cells stably transfected either with Six1 or control plasmids. D and E, Cell numbers (D) and percentage of BrdU positive cells (E) of Six1 siRNA and scramble control siRNA group in MIA PaCa-2 cells. F, Percentage of BrdU positive cells of Six1 siRNA and scramble control siRNA group in PANC-1 cells. All the experiments were performed in triplicate; bars, s.e.m.; *P<0.05.
(TIF)

Figure S2 **The effect of Six1 siRNAs on the endogenous expression levels of Six1 was examined in PANC-1 and MIA PaCa-2 cells by quantitative PCR (A and B) and western blot analyses (C and D).** GAPDH was used as an internal control. All the experiments were performed in triplicate; bars, s.e.m.; *P<0.05.
(TIF)

Figure S3 **Six1 correlates with cyclin D1 in the xenograft tumors from PANC-1 cells stably transfected with either Six1 or control plasmids as determined by western blot and then quantified by Fluorchem computer analysis (r = 0.721,** ***P*** **= 0.023).**
(TIF)

Figure S4 **Analyses of expression of various cyclins by Western blot in two stable cell lines ectopically overexpressing Six1: PANC-1 (A) and MIA PaCa-2 (B) cells.** GAPDH was used as an internal control.
(TIF)

## References

[1] Jemal A, Siegel R, Xu J, Ward E (2010) Cancer statistics, 2010. CA Cancer J Clin 60: 277–300. 20610543 10.3322/caac.20073

[2] Tempero MA, Berlin J, Ducreux M, Haller D, Harper P, et al (2011) Pancreatic cancer treatment and research: an international expert panel discussion. Ann Oncol 22: 1500–1506. 21199884 10.1093/annonc/mdq545

[3] Kumar JP (2009) The sine oculis homeobox (SIX) family of transcription factors as regulators of development and disease. Cell Mol Life Sci 66: 565–583. 18989625 10.1007/s00018-008-8335-4 PMC2716997

[4] Anderson AM, Weasner BM, Weasner BP, Kumar JP (2012) Dual transcriptional activities of SIX proteins define their roles in normal and ectopic eye development. Development 139: 991–1000. 22318629 10.1242/dev.077255 PMC3274360

[5] Ford HL, Kabingu EN, Bump EA, Mutter GL, Pardee AB (1998) Abrogation of the G2 cell cycle checkpoint associated with overexpression of HSIX1: a possible mechanism of breast carcinogenesis. Proc Natl Acad Sci U S A 95: 12608–12613. 9770533 10.1073/pnas.95.21.12608 PMC22878

[6] Zou D, Silvius D, Fritzsch B, Xu PX (2004) Eya1 and Six1 are essential for early steps of sensory neurogenesis in mammalian cranial placodes. Development 131: 5561–5572. 15496442 10.1242/dev.01437 PMC3882150

[7] Xu PX, Zheng W, Huang L, Maire P, Laclef C, et al (2003) Six1 is required for the early organogenesis of mammalian kidney. Development 130: 3085–3094. 12783782 10.1242/dev.00536 PMC3872112

[8] McCoy EL, Iwanaga R, Jedlicka P, Abbey NS, Chodosh LA, et al (2009) Six1 expands the mouse mammary epithelial stem/progenitor cell pool and induces mammary tumors that undergo epithelial-mesenchymal transition. J Clin Invest 119: 2663–2677. 19726883 10.1172/JCI37691 PMC2735909

[9] Laclef C, Souil E, Demignon J, Maire P (2003) Thymus, kidney and craniofacial abnormalities in Six 1 deficient mice. Mech Dev 120: 669–679. 12834866 10.1016/s0925-4773(03)00065-0

[10] Konishi Y, Ikeda K, Iwakura Y, Kawakami K (2006) Six1 and Six4 promote survival of sensory neurons during early trigeminal gangliogenesis. Brain Res 1116: 93–102. 16938278 10.1016/j.brainres.2006.07.103

[11] Reichenberger KJ, Coletta RD, Schulte AP, Varella-Garcia M, Ford HL (2005) Gene amplification is a mechanism of Six1 overexpression in breast cancer. Cancer Res 65: 2668–2675. 15805264 10.1158/0008-5472.CAN-04-4286

[12] Li CM, Guo M, Borczuk A, Powell CA, Wei M, et al (2002) Gene expression in Wilms’ tumor mimics the earliest committed stage in the metanephric mesenchymal-epithelial transition. Am J Pathol 160: 2181–2190. 12057921 10.1016/S0002-9440(10)61166-2 PMC1850829

[13] Yu Y, Khan J, Khanna C, Helman L, Meltzer PS, et al (2004) Expression profiling identifies the cytoskeletal organizer ezrin and the developmental homeoprotein Six-1 as key metastatic regulators. Nat Med 10: 175–181. 14704789 10.1038/nm966

[14] Ng KT, Man K, Sun CK, Lee TK, Poon RT, et al (2006) Clinicopathological significance of homeoprotein Six1 in hepatocellular carcinoma. Br J Cancer 95: 1050–1055. 17008870 10.1038/sj.bjc.6603399 PMC2360701

[15] Behbakht K, Qamar L, Aldridge CS, Coletta RD, Davidson SA, et al (2007) Six1 overexpression in ovarian carcinoma causes resistance to TRAIL-mediated apoptosis and is associated with poor survival. Cancer Res 67: 3036–3042. 17409410 10.1158/0008-5472.CAN-06-3755

[16] Imam JS, Buddavarapu K, Lee-Chang JS, Ganapathy S, Camosy C, et al (2010) MicroRNA-185 suppresses tumor growth and progression by targeting the Six1 oncogene in human cancers. Oncogene 29: 4971–4979. 20603620 10.1038/onc.2010.233

[17] Tan J, Zhang C, Qian J (2011) Expression and significance of Six1 and Ezrin in cervical cancer tissue. Tumour Biol 32: 1241–1247. 21874375 10.1007/s13277-011-0228-8

[18] Zheng XH, Liang PH, Guo JX, Zheng YR, Han J, et al (2010) Expression and clinical implications of homeobox gene Six1 in cervical cancer cell lines and cervical epithelial tissues. Int J Gynecol Cancer 20: 1587–1592. 21370601

[19] Coletta RD, Christensen KL, Micalizzi DS, Jedlicka P, Varella-Garcia M, et al (2008) Six1 overexpression in mammary cells induces genomic instability and is sufficient for malignant transformation. Cancer Res 68: 2204–2213. 18381426 10.1158/0008-5472.CAN-07-3141

[20] Coletta RD, Christensen K, Reichenberger KJ, Lamb J, Micomonaco D, et al (2004) The Six1 homeoprotein stimulates tumorigenesis by reactivation of cyclin A1. Proc Natl Acad Sci U S A 101: 6478–6483. 15123840 10.1073/pnas.0401139101 PMC404070

[21] Micalizzi DS, Christensen KL, Jedlicka P, Coletta RD, Barón AE, et al (2009) The Six1 homeoprotein induces human mammary carcinoma cells to undergo epithelial-mesenchymal transition and metastasis in mice through increasing TGF-beta signaling. J Clin Invest 119: 2678–2690. 19726885 10.1172/JCI37815 PMC2735914

[22] Wang CA, Jedlicka P, Patrick AN, Micalizzi DS, Lemmer KC, et al (2012) SIX1 induces lymphangiogenesis and metastasis via upregulation of VEGF-C in mouse models of breast cancer. J Clin Invest 122: 1895–1906. 22466647 10.1172/JCI59858 PMC3336979

[23] Ng KT, Lee TK, Cheng Q, Wo JY, Sun CK, et al (2010) Suppression of tumorigenesis and metastasis of hepatocellular carcinoma by shRNA interference targeting on homeoprotein Six1. Int J Cancer 127: 859–872. 20013809 10.1002/ijc.25105

[24] Schmittgen TD, Livak KJ (2008) Analyzing real-time PCR data by the comparative C(T) method. Nat Protoc 3: 1101–1108. 18546601 10.1038/nprot.2008.73

[25] Rhodes DR, Yu J, Shanker K, Deshpande N, Varambally R, et al (2004) ONCOMINE: a cancer microarray database and integrated data-mining platform. Neoplasia 6: 1–6. 15068665 10.1016/s1476-5586(04)80047-2 PMC1635162

[26] Luo X, Li Z, Li X, Wang G, Liu W, et al (2011) hSav1 interacts with HAX1 and attenuates its anti-apoptotic effects in MCF-7 breast cancer cells. Int J Mol Med 28: 349–355. 21567072 10.3892/ijmm.2011.692

[27] Zhang G, Schetter A, He P, Funamizu N, Gaedcke J, et al (2012) DPEP1 inhibits tumor cell invasiveness, enhances chemosensitivity and predicts clinical outcome in pancreatic ductal adenocarcinoma. PLoS One 7: e31507. 22363658 10.1371/journal.pone.0031507 PMC3282755

[28] Badea L, Herlea V, Dima SO, Dumitrascu T, Popescu I (2008) Combined gene expression analysis of whole-tissue and microdissected pancreatic ductal adenocarcinoma identifies genes specifically overexpressed in tumor epithelia. Hepatogastroenterology 55: 2016–2027. 19260470

[29] Donahue TR, Tran LM, Hill R, Li Y, Kovochich A, et al (2012) Integrative survival-based molecular profiling of human pancreatic cancer. Clin Cancer Res 18: 1352–1363. 22261810 10.1158/1078-0432.CCR-11-1539 PMC3816537

[30] Luo X, Li Z, Yan Q, Li X, Tao D, et al (2009) The human WW45 protein enhances MST1-mediated apoptosis in vivo. Int J Mol Med 23: 357–362. 19212654 PMC2851627

[31] Laurance ME, Starr DB, Michelotti EF, Cheung E, Gonzalez C, et al (2001) Specific down-regulation of an engineered human cyclin D1 promoter by a novel DNA-binding ligand in intact cells. Nucleic Acids Res 29: 652–661. 11160886 10.1093/nar/29.3.652 PMC30392

[32] Ishikawa M, Yoshida K, Yamashita Y, Ota J, Takada S, et al (2005) Experimental trial for diagnosis of pancreatic ductal carcinoma based on gene expression profiles of pancreatic ductal cells. Cancer Sci 96: 387–393. 16053509 10.1111/j.1349-7006.2005.00064.x PMC11160054

[33] Buchholz M, Braun M, Heidenblut A, Kestler HA, Klöppel G, et al (2005) Transcriptome analysis of microdissected pancreatic intraepithelial neoplastic lesions. Oncogene 24: 6626–6636. 16103885 10.1038/sj.onc.1208804

[34] Pei H, Li L, Fridley BL, Jenkins GD, Kalari KR, et al (2009) FKBP51 affects cancer cell response to chemotherapy by negatively regulating Akt. Cancer Cell 16: 259–266. 19732725 10.1016/j.ccr.2009.07.016 PMC2755578

[35] Yu Y, Davicioni E, Triche TJ, Merlino G (2006) The homeoprotein six1 transcriptionally activates multiple protumorigenic genes but requires ezrin to promote metastasis. Cancer Res 66: 1982–1989. 16488997 10.1158/0008-5472.CAN-05-2360

[36] Liu Y, Nandi S, Martel A, Antoun A, Ioshikhes I, et al.. (2012) Discovery, optimization and validation of an optimal DNA-binding sequence for the Six1 homeodomain transcription factor. Nucleic Acids Res. 10.1093/nar/gks587 PMC3458543 22730291

[37] Abate-Shen C (2002) Deregulated homeobox gene expression in cancer: cause or consequence? Nat Rev Cancer 2: 777–785. 12360280 10.1038/nrc907

[38] Malumbres M, Barbacid M (2009) Cell cycle, CDKs and cancer: a changing paradigm. Nat Rev Cancer 9: 153–166. 19238148 10.1038/nrc2602

[39] Musgrove EA, Caldon CE, Barraclough J, Stone A, Sutherland RL (2011) Cyclin D as a therapeutic target in cancer. Nat Rev Cancer 11: 558–572. 21734724 10.1038/nrc3090

