# Supplementary figures and images for: Six1 Promotes Proliferation of Pancreatic Cancer Cells via Upregulation of Cyclin D1 Expression
Source: PLoS One. 2013 Mar 20;8(3):e59203. doi: 10.1371/journal.pone.0059203 (PMC3604102; doi:10.1371/journal.pone.0059203)

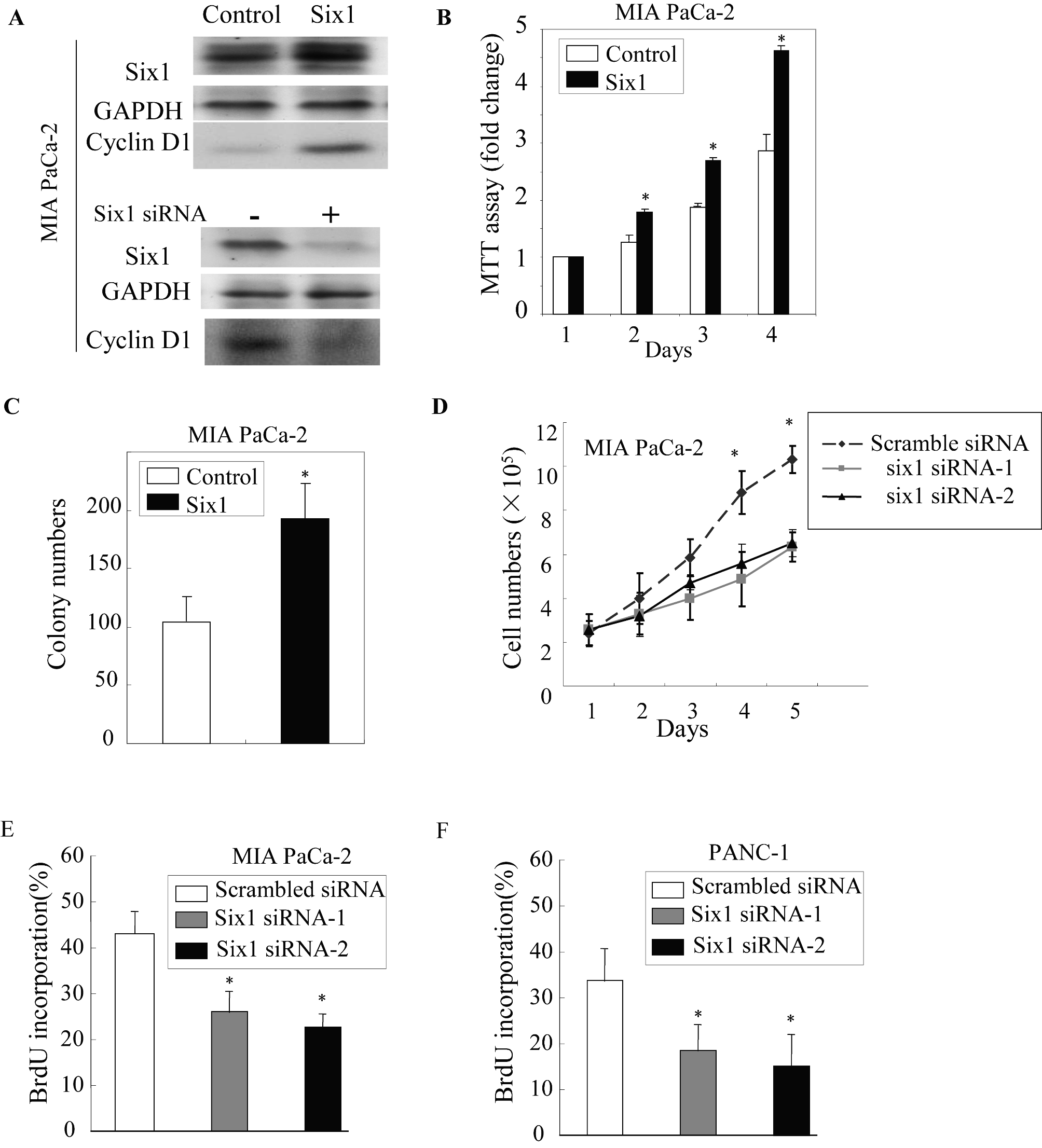

Supplement: Figure S1 — Six1 promotes the growth of PANC-1 and MIA PaCa-2 cells. A, Western blot analysis of the expression of Six1 and cyclin D1 in MIA PaCa-2 cells. B and C, MTT growth assays and colony formation assay of the cells stably transfected either with Six1 or control plasmids. D and E, Cell numbers (D) and percentage of BrdU positive cells (E) of Six1 siRNA and scramble control siRNA group in MIA PaCa-2 cells. F, Percentage of BrdU positive cells of Six1 siRNA and scramble control siRNA group in PANC-1 cells. All the experiments were performed in triplicate; bars, s.e.m.; *P<0.05. (TIF) [file pone.0059203.s001.tif]

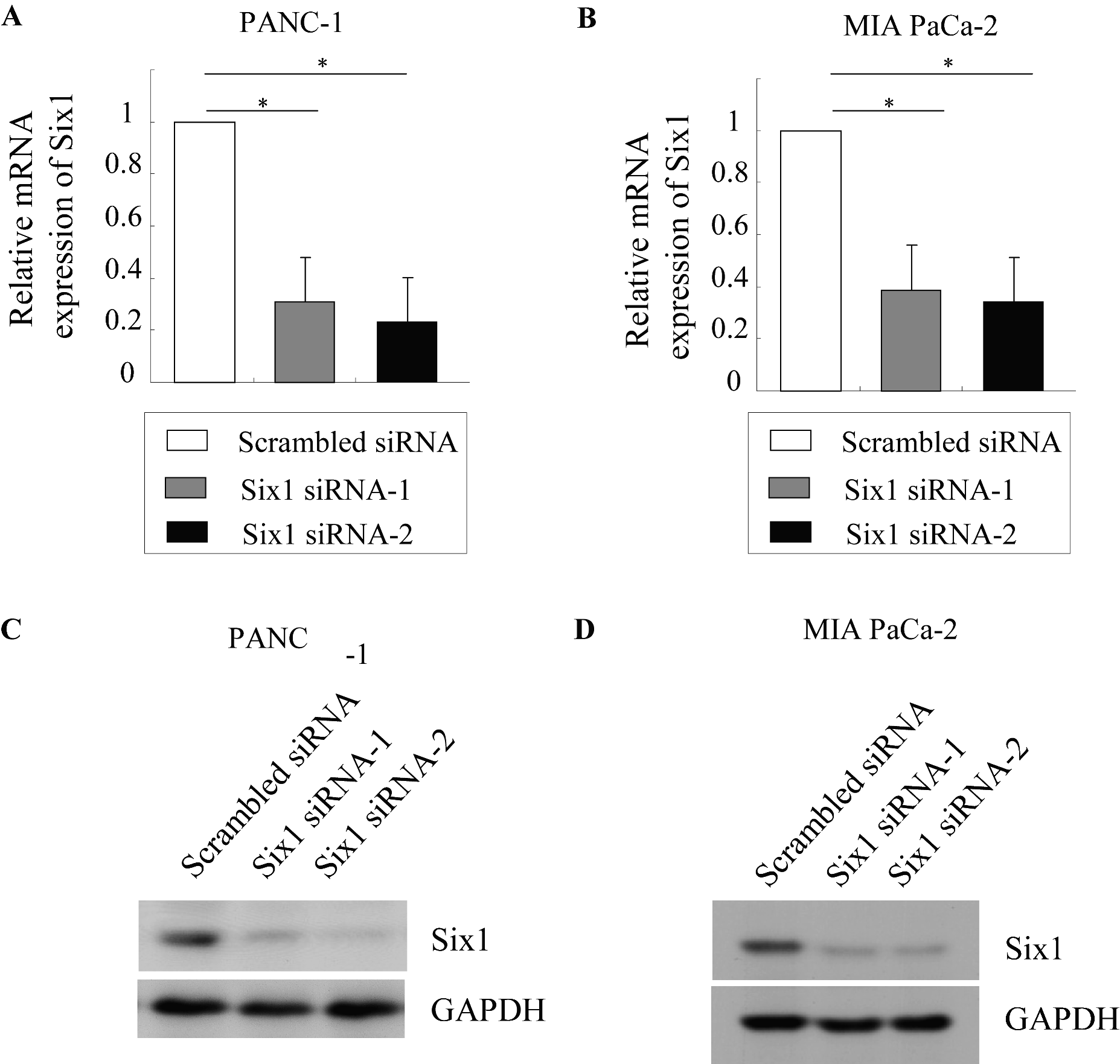

Supplement: Figure S2 — The effect of Six1 siRNAs on the endogenous expression levels of Six1 was examined in PANC-1 and MIA PaCa-2 cells by quantitative PCR (A and B) and western blot analyses (C and D). GAPDH was used as an internal control. All the experiments were performed in triplicate; bars, s.e.m.; *P<0.05. (TIF) [file pone.0059203.s002.tif]

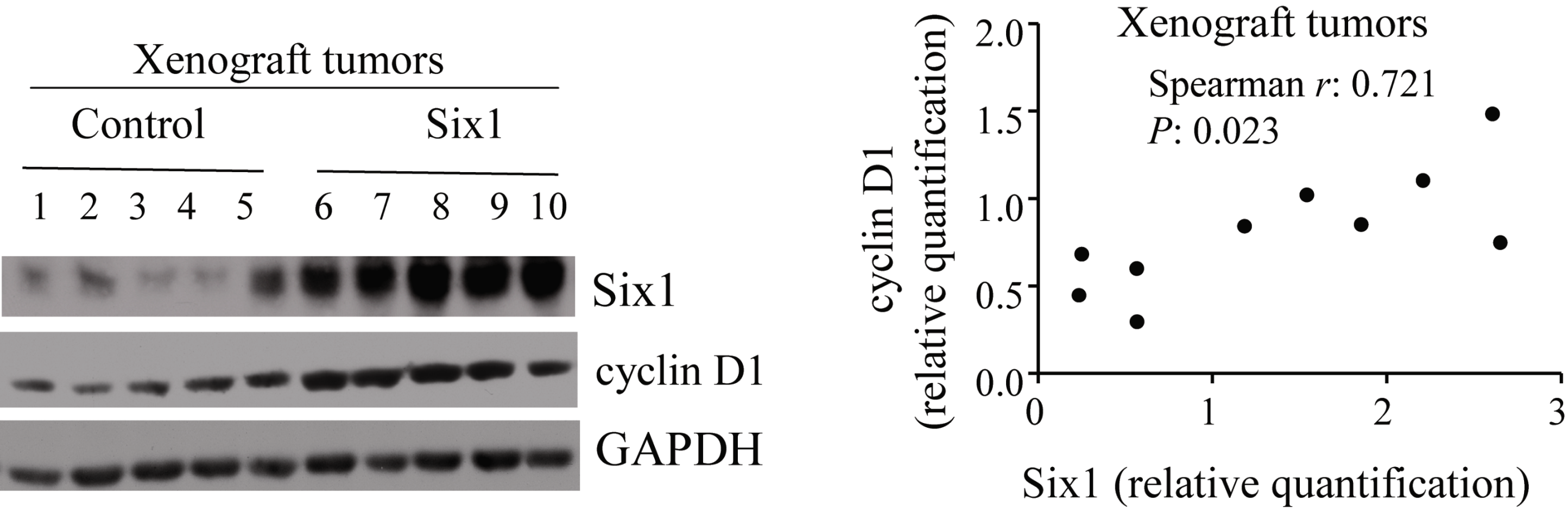

Supplement: Figure S3 — Six1 correlates with cyclin D1 in the xenograft tumors from PANC-1 cells stably transfected with either Six1 or control plasmids as determined by western blot and then quantified by Fluorchem computer analysis (r = 0.721, P = 0.023). (TIF) [file pone.0059203.s003.tif]

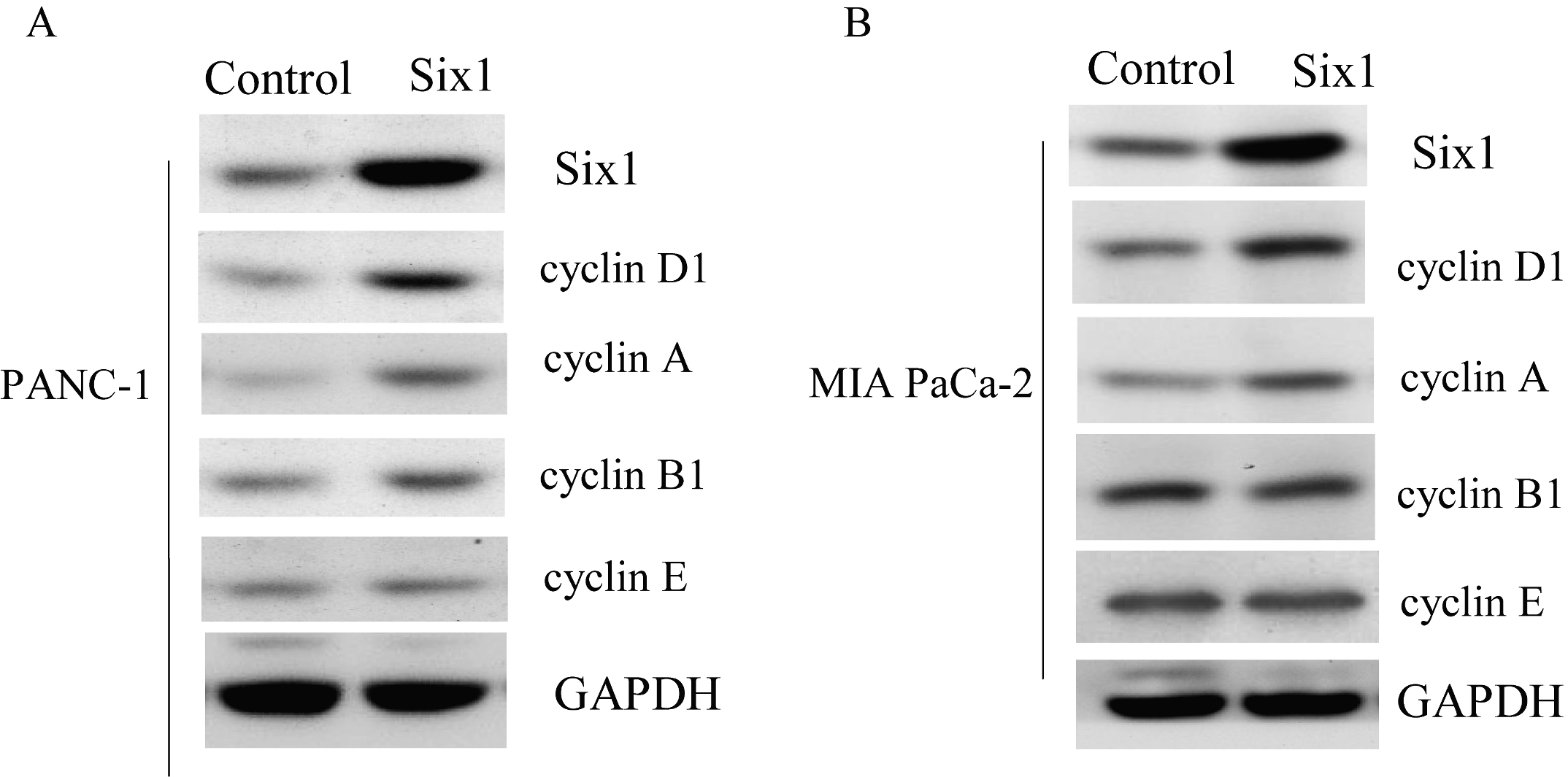

Supplement: Figure S4 — Analyses of expression of various cyclins by Western blot in two stable cell lines ectopically overexpressing Six1: PANC-1 (A) and MIA PaCa-2 (B) cells. GAPDH was used as an internal control. (TIF) [file pone.0059203.s004.tif]
